# Drug-induced prolonged corrected QT interval in patients with methadone and opium overdose

**DOI:** 10.1186/s13011-019-0196-3

**Published:** 2019-02-20

**Authors:** Davood Soroosh, Mahbubeh Neamatshahi, Bahram Zarmehri, Samaneh Nakhaee, Omid Mehrpour

**Affiliations:** 1 Department of forensic medicine, Sabzevar university of medical sciences, Sabzevar, Iran; 2 Department of social medicine. Faculty of medicine, Sabzevar university of medical sciences, Sabzevar, Iran; 3 Department of emergency medicine, faculty of medicine, Mashhad university of medical sciences, Mashhad, Iran; 4 Medical Toxicology and Drug Abuse Research Center (MTDRC), Birjand University of Medical Sciences, Birjand, Iran; 5 Rocky Mountain Poison and Drug Center, Denver, CO USA

**Keywords:** Corrected QT interval, Methadone, Opium, Overdose

## Abstract

**Background:**

Iran is a country with the highest rate of opioid addiction in the world. The most commonly used opioid in Iran is opium, and methadone is in second place. The trend of drug use has changed from opium to methadone from 2006 to 2011. Presence of a large number of addicted people and methadone maintenance therapy clinics make methadone readily available in Iran. Therefore, evaluation of the epidemiological characteristic of methadone toxicity and its effects on the heart is essential.

**Methods:**

In This cross-sectional, retrospective, descriptive, analytical study all patients with methadone or opium toxicity who had been admitted to Vasei hospital, Sabzevar, Iran, during the years 2015 and 2016 were included, and their records were evaluated. Demographic data, addiction history, underlying diseases, and the outcome of admission were recorded. Then, corrected QT interval **(**QTc) of the first ECG of the patients after admission was evaluated.

**Results:**

The Majority of toxicities occurred in those above 30 years of age (71.4%), who lived in cities (62.8%), and were married (69.2%). A positive history of addiction was considerably higher in the opium group (72.3% versus 43.3%). There was no significant difference regarding QTc prolongation between patients with methadone and opium toxicity (*p* = 0.3).

**Conclusion:**

QTc prolongation is one of the adverse effects of methadone or opium overdose. It seems that significant QTc prolongation is not uncommon among patients with opium overdose.

## Introduction

Iran is a country with the highest rate of opioid addiction in the world [1, 2]. Eastern Iran shares a border with Afghanistan where the majority of opium in the world is produced. Iran is the major route for drug transport to Europe [3]. It is estimated that there are about 2 million drug users in Iran that 9 to16 percent of them are intravenous drug users in Iran [4, 5]. The World Health Organization (WHO) reported that Iran has opium consumption three times greater than the mean of the world. The most commonly used opioid in Iran is opium (82%). Methadone is the third most common used opioid with a prevalence of 16.6% of opioid use. There are around 5000 outpatient buprenorphine or methadone maintenance therapy (MMT) clinics for the sole purpose of the treatment of opioid dependency in Iran and covered about 500,000 people for treatment [6]. In a study in Tehran, the capital city of Iran, narcotics were the second common cause of death (24.75%), and opium was the most prevalent drug of use. The trend of drug use has changed over recent years. Opium, commonly used in early 2006, was replaced by methadone by the later part of 2011 [7]. Methadone poisoning can occur accidentally or intentionally. Overdose or use in children or elderly is usually accidental whereas suicide or homicide attempts are intentional [8]. Methadone poisoning is common in Iran due to a large number of addicted people under MMT protocol, which results in the availability of methadone to other family members and friends [9, 10].

Some medications like cocaine and methadone may cause QTc prolongation [11, 12]. Prolonged QTc is not uncommon among patients on methadone maintenance therapy [1, 13]. In cases with severe prolonged QTc (more than 500 ms), life-threatening dysrhythmias like torsade de pointes (TdP) may be induced [14, 15]. TdP may potentially degenerate into ventricular fibrillation and cause sudden cardiac death, if not treated promptly [16]. So, prolonged QTc can be associated with all-cause mortality, cardiovascular death and sudden cardiac death [17]. Very few studies evaluated the effect of opium on QTc interval. Also, limited studies assessed QTc prolongations in toxicity of methadone in comparison to another common opioid toxicity (opium overdose).

Because methadone is readily available in Iran and the number of individuals undergoing MMT is increasing every day, it seems that there is a need for an epidemiological study of this toxicity to improve the quality of care and formulate prevention plans. Therefore, the epidemiological characteristics of methadone toxicity and effects of methadone on QTc interval were evaluated in this study and compared with opium toxicity.

## Material &Methods

This was a cross-sectional, retrospective, descriptive, analytical study. The study was approved by the ethical committee of Sabzevar University of Medical Sciences (code number: IR.MEDSAB.REC.1396.77).

All patients with methadone or opium toxicity admitted to the Vasei hospital, Sabzevar, Iran, during the years 2015 and 2016, with toxicity confirmed by urine screen test, were included in this study. Initially, the records of all patients with methadone or opium toxicity were obtained. Patients with drug toxicities other than methadone or opium alone were not included in the study. Also, patients with a history of cardiac dysrhythmia or electrolyte imbalance were excluded. Standard treatments such as oxygen therapy, hydration, supportive cares, naloxone therapy (in case of respiratory depression) according to *Goldfrank’s Toxicologic* Emergencies textbook, Tenth Edition, was performed for patients if indicated. Also, patients with severe QTc prolongation were treated with magnesium sulfate [18, 19].

The demographic data including age, gender, marital status, residency, addiction history, underlying diseases, and the outcome of admission were recorded. Then, the initial ECG of patients underwent blind evaluation by one of the researchers. Presence of any dysrhythmias or including prolonged QT interval and QTc (QT interval corrected with heart rate by bazett’s formula: QTc = QT/√RR) prolongation were evaluated. A QT > 450 ms and QTc > 470 ms were considered as prolonged [20]. A *p*-value less than 0.05 were considered significant. The statistical analysis was performed using SPSS version 16. Using the Kolmogorov-Smirnov test, we also examined the normality of quantitative variables. Statistical tests including Chi-square and independent –T-test were used.

## Results

Out of 234 patients, 97(41.45%) had methadone toxicity, and 137(58.57%) had opium toxicity. In the methadone and opium groups, 66(68%) and 88(64%) patients were male, respectively. Majority of toxicities occurred in patients above 30 years old (*n* = 167; 71.4%) married (69.2%) and who are living in cities (62.8%), Tables 1 and 2 show comparisons of some demographic and clinical variables and the most common underlying diseases in the methadone and opium groups, respectively.

**Table 1:** Comparison of demographic and clinical information between methadone and opium groups

		Frequency (percent) in methadone group	Frequency (percent) in opium group	Total frequency (percent)	*p*- value
Age	< 30	39 (40.2%)	28 (20.4%)	67 (28.6%)	*p* = 0.001
	≥30	58 (59.8%)	109 (79.6%)	167 (71.4%)	
Residence	City	67 (69.1%)	80 (54.8%)	147 (62.8%)	*p* = 0.09
	Village	30 (30.9%)	57 (46.1%)	87 (37.2%)	*p* = 0.001
Marital status	Married	62 (63.9%)	100 (73%)	162 (69.2%)	*p* = 0.13
	Single, widow, or divorced	35 (36.1%)	37 (27%)	72 (30.8%)	
Positive history of addiction		42 (43.3%)	99 (72.3%)	141 (60.3%)	*p* < 0.0001
Mortality		1 (1%)	3 (2.2%)	4 (2%)	*p* = 0.5
Smoking history		45 (46.4%)	56 (40.9%)	101 (43.2%)	*p* = 0.4
QTc prolongation		53 (54.6%)	83 (60.6%)	136 (58.1%)	*p* = 0.3

Comparisons were made using chi- square test

**Table 2:** Comparison of most common underlying conditions in the methadone and opium groups

Underlying disease	Frequency (percent) in methadone group	Frequency (percent) in opium group	Total frequency (percent)
Hypertension	14 (14.4%)	25 (18.2%)	39 (17%)
Diabetes	7 (7.2%)	21 (15.4%)	28 (12%)
Hyperlipidemia	6 (6.2%)	8 (5.8%)	14 (6%)
Ischemic heart disease	7 (7.2%)	10 (7.3%)	17 (7%)
No underlying disease	63 (65%)	73 (53.3%)	136 (58%)
Total	97 (100%)	137 (100%)	234 (100%)

The mean QTc intervals were 482 ± 76 ms and 483 ± 65 ms in the methadone and opium groups, respectively. The mean QTc prolongations were 53 ms and 83 ms in the methadone and opium groups, respectively. The mean QT intervals were 420 ± 62 ms and 427 ± 51 ms in the methadone and opium groups, respectively. 54.6 and 60.6% of patients with methadone and opium overdose had QTc prolongation, respectively. Based on the results of the Chi-square test, there was no significant difference regarding QTc prolongation between patients with methadone and opium toxicity (*p* = 0.3). QT interval of greater than 450 ms in methadone and opium group was 29% and 27%, respectively the mean QTc intervals in the male and female groups were 480 ± 81 and 487 ± 66, respectively. The Chi-square test showed, there were no significant differences in the QTc prolongation between male and female (*p* = 0.4).

No case of torsade de pointes was observed in this study. Based on the Chi-square test there were no significant differences regarding the positive history of smoking between methadone and opium groups (*p* = 0.4). The Chi-square test showed there were significant differences regarding a positive history of addiction between the two groups (72.3% versus 43.3%, *p* < 0.0001).

## Discussion

According to the results of this study, QTc prolongation was observed in 54.6 and 60.6% of patients with methadone and opium toxicity, and there was not a statically significant difference regarding QTc prolongation between two groups.

Farsi et al. in a prospective cross-sectional study concluded that QTc more than 450 ms was observed in 50.7% methadone poisoned patients. Some dangerous outcomes like ICU admission, intubation and death are correlated with QTc interval in patients with acute methadone overdose [21] . In a recent study, 39.2% of patients who had died with a confirmed diagnosis of pure methadone toxicity had on-presentation QTc prolongation [22]. A study conducted in Tehran demonstrated that methadone was the most common cardiotoxic drug [23].

Westermeyer et al. reported QTc prolongation among 34% of the patients on MMT over 4 years [24]. A 2011 study examining 155 patients under MMT it was found, only 18.1% had QTc prolongation, with no report of torsade de pointes [21]. In a survey in 2012, QTc prolongation rate was reported to be 11.1% in 180 MMT patients [21]. Consistent with this study, others reported that most observed ECG abnormalities in opiate addicts,were ST abnormalities (19%), QTc prolongation (13%), tall R- and/or S-waves (11%) and missing R progression (10%). [25].

The wide difference between the results of this study and that of the cited studies may be because the patients in some of the cited studies were stable and on MMT and the patients in this study had methadone toxicity or overdose.

A study published in 2003, was demonstrated that the QTc interval is positively related to the methadone dose [21]. A systematic review published in 2015 on the cardiotoxicity of methadone noted inconsistencies in the correlation of methadone dose with QTc prolongation and torsade de pointes [1]. Several large studies have been performed to evaluate the relationship between the dose of methadone and QTc prolongation. Some have reported a direct relationship [13, 26] while others could not find such an association [27–29]. An experimental study suggests that methadone blocks the rapid component of the delayed rectifier potassium current (IKr) in a dose-dependent manner [30]. This is a common mechanism through which drug-induced QT prolongation and torsade de pointes is mediated [26].

Interestingly, 60.6% of patients with opium toxicity had QT prolongation. To our knowledge, there are limited studies in humans on the effect of opium overdose on QT interval. Rismantab-Sani (2017) reported that QT interval prolongation (4.6%) was the most common ECG changes in patients with acute opium overdose. Mechanistically, no study evaluated the effect of opium on rapid delayed rectifier K+ current (IKr) conducted by hERG channels. So further studies are needed to evaluate mechanism through opium-induced QT prolongation [31]. In an animal study, Najafipour and Joukar evaluated the long-term and short-term effects of opium smoking in association with hypercholesterolemic diet on the incidence of cardiac dysrhythmias. They showed that short term opium smoking along with hypercholesterolemia significantly increased QTc intervals. However, in long-term opium groups, QTc did not change significantly compared to their controls [32]. In some studies, opium addiction is considered as a risk factor in the emergence of ventricular dysrhythmia after acute myocardial infarction [33–36]. The initial pathophysiology of dysrhythmia is directly related to the presence of dysfunctions in the conduction system. The opioid receptors in the atria and ventricles may play an important role in the development of different dysrhythmias [37, 38]. Evidence shows that k opioid receptors can be involved in the development of a dysrhythmic response. Coles et al. showed that the activation of k opioid receptors in pigs is prodysrhythmic [39]. Some studies state that the potential dysrhythmogenic activities of opioids in small doses occur through activation of k opioid receptors and their anti-dysrhythmic actions in larger doses happen due to direct interaction with the heart cell membrane [40].

In this study, the most prevalent underlying diseases with QTc prolongation were hypertension and ischemic heart diseases observed. ECG is usually advised for patients on MMT with concomitant heart or liver disease, electrolyte abnormalities, or QTc-prolonging medications [21]. In agreement with this study, Najafipour and Joukar (2012) showed a trend of increasing incidence of dysrhythmia during myocardial ischemia in rabbits exposed to opium smoke, especially those with hypercholesterolemia [32].

Based on the results of this study regarding place of residence, patients with opium overdose were significantly villagers in comparison to another group. Previous study suggested that villagers were more likely to be opium users [41] For poor villagers, with little access to doctors or health systems, opium could be considered as a “miracle” drug of the countryside [42]. Besides, no significant difference in the QTc prolongation between males and females observed. This could be because the different upper limits for QTc for males and females were not considered separately.

This study had some limitations. The upper limit of the QTc interval is different among males and females, but this was not considered separately. Patients with a history of cardiovascular disease were enrolled and this may cause a confounding effect. Additionally, performing a urine drug screen for methadone and opium has limitations. The screen may only show exposure to these agents in recent days and does not represent their serum levels. Serum toxicity profiles, including opioid concentrations, were unable to be tested. Therefore, a correlation between opioid concentrations and severity of QTc prolongation could not be determined. Also, in this study, a control group comparison was not included that could be considered in future studies. The underlying mechanism of opium-induced QTc prolongation even warranted more studies.

## Conclusion

It seems that significant QTc prolongation is not uncommon among patients with opium and methadone over dose, and there were not statically substantial differences regarding QTc prolongation between two groups QTc prolongation is one of the adverse effects of methadone or opium overdose, and it may be more prevalent among patients with a history of hypertension and/or ischemic heart disease. The results of this study can be helpful in the exchange of ideas, for reducing the harms from substance overdose. High-risk individuals can be motivated to avoid drug use by educating them about its harmful effects. And policymakers can provide prevention programs. So in patients with methadone or opium poisoning integrating cardiac dysrhythmia risk assessment and performance of electrocardiography into routine care process can be necessary for preventing clinically significant outcomes (life-threatening dysrhythmias or TdP). The utility of routinely monitoring these patients for prolonged QTc even when they receive additional QT prolonging drugs is warranted for future studies.

## Abbreviations

ECG: Electrocardiography
MMT: Methadone maintenance therapy
QTc: Corrected QT
WHO: World Health Organization
